# Response of Nagpur mandarin (*Citrus reticulata* Blanco) to high density planting systems

**DOI:** 10.1038/s41598-021-89221-4

**Published:** 2021-05-25

**Authors:** M. S. Ladaniya, R. A. Marathe, A. A. Murkute, A. D. Huchche, A. K. Das, Anjitha George, Jayashree Kolwadkar

**Affiliations:** 1 ICAR-Central Citrus Research Institute, Nagpur, Maharashtra 440033 India; 2 Present Address: ICAR-National Bureau of Soil Survey and Land Use Planning, Nagpur, 440033 India

**Keywords:** Plant sciences, Plant development, Plant physiology

## Abstract

High density planting system i.e. accommodating a higher number of plants than routine in a given area is an innovative agro-technology to increase yield and thereby early net returns. Due to conventional wide spacing plantation in Nagpur mandarin (*Citrus reticulata* Blanco), the land remains unutilized as the plant canopy gradually increases over the years. In the present study, Nagpur mandarin (*Citrus reticulata* Blanco) budded on Rangpur lime rootstock was evaluated under six different planting spacings. It was observed that the organic carbon (1.10–1.82%) and major nutrients viz. N (309–430 kg ha^−1^), P (20–54 kg ha^−1^) and K (291–810 kg ha^−1^) increased vis-à-vis plant density and was highest under 2 × 2 m spacing. Plants were tallest at 2 × 2 m spacing with the higher PAR interception (88.2) and the lowest leaf area index (1.09). Fruit yield on area basis, under 2 × 2 m spacing was 26, 7.1, 4.6 times more as compared to conventional plantation during the first, second and third year, respectively. At fifth year of crop harvest, the highest B:C ratio (6.36) was recorded in 6 × 3 m followed by 4 × 2 m and 2 × 2 m.

## Introduction

Mandarins (*Citrus reticulata* Blanco) are easily peelable fruits and segments are conveniently consumed by hands. Among citrus group, mandarins contributes to the second largest production (26%) after sweet oranges (56%) in the world citrus basket^1^. Out of total 12.51 million tonnes of citrus production in India, mandarins constitutes 5.27 million metric tonnes from 0.42 million ha area and ranks the first among the citrus fruits grown in the country^2^. The average national productivity of mandarins in India is 12.54 tonnes ha^−1^, which is fairly low as compared to many advanced mandarin growing countries. Mandarin cultivation is popular among citrus growers due to its constant demand in the domestic market and easy adaptability to varied agro-climatic conditions. Among mandarins, “Nagpuri” or “Nagpur” mandarin is cherished for its unique thirst quenching sweet and sour taste. The Vidarbha region of Maharashtra (a major pocket of Nagpur mandarin in Maharashtra) and adjoining parts of Madhya Pradesh and Rajasthan (Jhalawar district) have more or less similar agro-climatic conditions and hence cultivation of this mandarin cultivar is blooming and expanding in these areas. The low productivity of mandarins in these regions is primarily attributed to senile old orchards, conventional wide spacing (6 × 6 m) and poor orchard management. This scenario demands innovative horticultural practices to get high and early returns for investments, particularly in initial years of orchard establishment.

Often due to wide spacing and low canopy volume, the spacing of 6 × 6 m fails to harness the available land during the initial phases of orchard development. This has given the thrust to evaluate the concept of high density planting (HDP) and ultra-high density planting (UHDP) for increasing the production and returns per unit area. It is the concept of HDP to exploit vertical and horizontal cropping area, to reap maximum profit against invested inputs and natural resources. The HDP and UHDP not only provide initial high production and net returns, especially during first 10–15 years, but also facilitates efficient use of fertilizers, irrigation and other inputs^3–6^. The main advantages of these intensive systems of cropping are precocity, low cost per unit production, possibility of higher mechanization, automation as in fertigation with higher input use efficiency. In commercial plantations, mostly smaller canopies are obtained either by using dwarfing rootstocks or by training and pruning (canopy management practices). For Nagpur mandarin and acid lime cv. ‘Kagzi’, dwarfing rootstocks like Flying dragon (*Poncirus trifoliata*) and hybrids like Troyer and Carrizo citranges were evaluated in long term trials at ICAR—Central Citrus Research Institute, Nagpur, India. However, these rootstocks with *Poncirus trifoliata* parentage did not perform satisfactorily^7–9^. In HDP and UHDP, light intensity may reduce to as low as 2% due to overcrowding of trees if not pruned^10^. It also increases the insect-pest and disease infestation and pose difficulties in cultural practices. Although high density planting means growing more number of plants as compared to conventional planting density, the exact limit of plant density need to be decided based on several factors including benefit: cost ratio.

The high density planting studies in Nagpur mandarin are very limited and without any concrete technology package. Further, there are no reports on UHDP in Nagpur mandarin. Therefore, to evaluate HDP and UHDP system, understand physiological performance and develop a package of practices, present investigation was planned for Nagpur mandarin raised on Rangpur lime rootstock, a commercially successful rootstocks, under agro-climatic conditions of central India at Nagpur.

## Results and discussion

### Soil fertility status

Different planting treatments significantly influenced the soil organic carbon content buildup and was higher than pre-experiment status (Table 1). High content of organic carbon (1.48–1.82%) recorded under UHDP-2 as compared to conventional 6 × 6 m spacing (1.10–1.34%) was significant. There was substantial increase during initial years and it stabilized afterwards. High density planting covers the soil under the tree canopy and does not let it expose to sun light. It reduces the photodecomposition triggered loss of soil organic carbon. The decreasing organic carbon content with the increasing plant spacing could be attributed to the exposure of more soil surface to sunrays in wider spacings^6^. It takes about 3 years to oxidize 2–14% of organic carbon in soils due to sunlight^11^. Addition of vermicompost for better growth and balanced nutrition to every plant and decomposition of substantial quantity of organic matter could have contributed to increasing the organic carbon of the soil^12,13^ as the manures themselves contribute partially in organic carbon buildup under high density plantings.

**Table 1:** Effect of planting density on soil fertility status.

Treatments	2015–16	2016–17	2017–18	2018–19	2019–20
**Organic carbon (%)**					
Control	1.10^c^	1.21^c^	1.26^c^	1.30^b^	1.34^d^
HDP-1	1.28^abc^	1.42^b^	1.54^b^	1.60^a^	1.64^c^
HDP-2	1.25^bc^	1.48^a^	1.52^b^	1.65^a^	1.68^bc^
UHDP-1	1.35^ab^	1.50^a^	1.64^ab^	1.72^a^	1.75^ab^
UHDP-2	1.48^a^	1.58^a^	1.70^a^	1.80^a^	1.82^a^
**Available nitrogen (kg ha**^**−1**^**)**					
Control	309.3^b^	341.9^c^	363.7^b,c^	385.2^c^	380.4^c^
HDP-1	325.0^a,b^	366.0^b^	353.7^c,d^	374.1^d^	382.1^c^
HDP-2	310.0^b^	375.4^a,b^	342.0^d^	365.7^e^	370.1^c^
UHDP-1	332.1^a,b^	383.0^a^	379.2^b^	401.9^b^	405.3^b^
UHDP-2	347.7^a^	399.0^a^	402.9^a^	422.9^a^	430.1^a^
**Available phosphorus (kg ha**^**−1**^**)**					
Control	21.5^b^	32.8^b^	33.1^d^	34.1^c^	34.5^c^
HDP-1	22.1^b^	42.8^a,b^	42.6^b,c^	44.0^b^	45.6^b^
HDP-2	20.4^b^	40.5^a,b^	41.1^c^	42.6^b^	46.4^b^
UHDP-1	23.6^b^	44.8^a^	45.3^b^	46.0^a,b^	48.7^a,b^
UHDP-2	26.6^a^	48.9^a^	49.5^a^	51.1^a^	54.6^a^
**Available potassium (kg ha**^**−1**^**)**					
Control	291.9^c^	504.6^c^	495.2^c^	715.4^b^	720.1^c^
HDP-1	315.0^b^	542.3^b^	563.6^b^	800.8^a^	810.2^a^
HDP-2	310.1^b,c^	510.4^c^	572.8^b^	621.3^c^	628.7^e^
UHDP-1	330.0^a,b^	560.2^a,b^	596.4^a^	681.8^b^	690.2^d^
UHDP-2	345.1^a^	574.3^a^	575.8^a,b^	770.0^a^	775.4^b^

Means with at least one letter common are not statistically significant using TUKEY's Honest Significant Difference at 5%

Control: Planting at 6 m (row to row) × 6 m (plant to plant) spacing (tree density—277 plants ha^−1^), HDP-1: Planting at 6 m (row to row) × 3 m (plant to plant) spacing (tree density—555 plants ha^−1^), HDP-2: Planting at 4 m (row to row) × 4 m (plant to plant) spacing (tree density—625 plants ha^−1^), UHDP-1: Planting at 4 m (row to row) × 2 m (plant to plant) spacing (tree density—1250 plants ha^−1^), UHDP-2: Planting at 2 m (row to row) × 2 m (plant to plant) spacing (tree density—2500 plants ha^−1^).

During progressing years, available nutrient contents of soil i.e. N, P and K significantly increased amongst the treatments from 309.3 to 430.1, from 20.4 to 54.6 and from 291.9 to 810.2 kg ha^−1^, respectively (Table 1). Although K availability was excess in all the treatments, N and P were found to be in sufficiency range over the experimentation period. Higher contents of all the macronutrients were found in high density systems as compared to control. N and P released through the sufficiently available organic manures’ decomposition in high density planting could have increased N and P contents. The increased availability of P could also be linked to the acidulating effect of vermicompost on applied and native P. The higher K in all the treatments might have sourced from potash rich black clay soils formed on Deccan plateau of central India^14^.

### Plant growth

**Figure 1:**
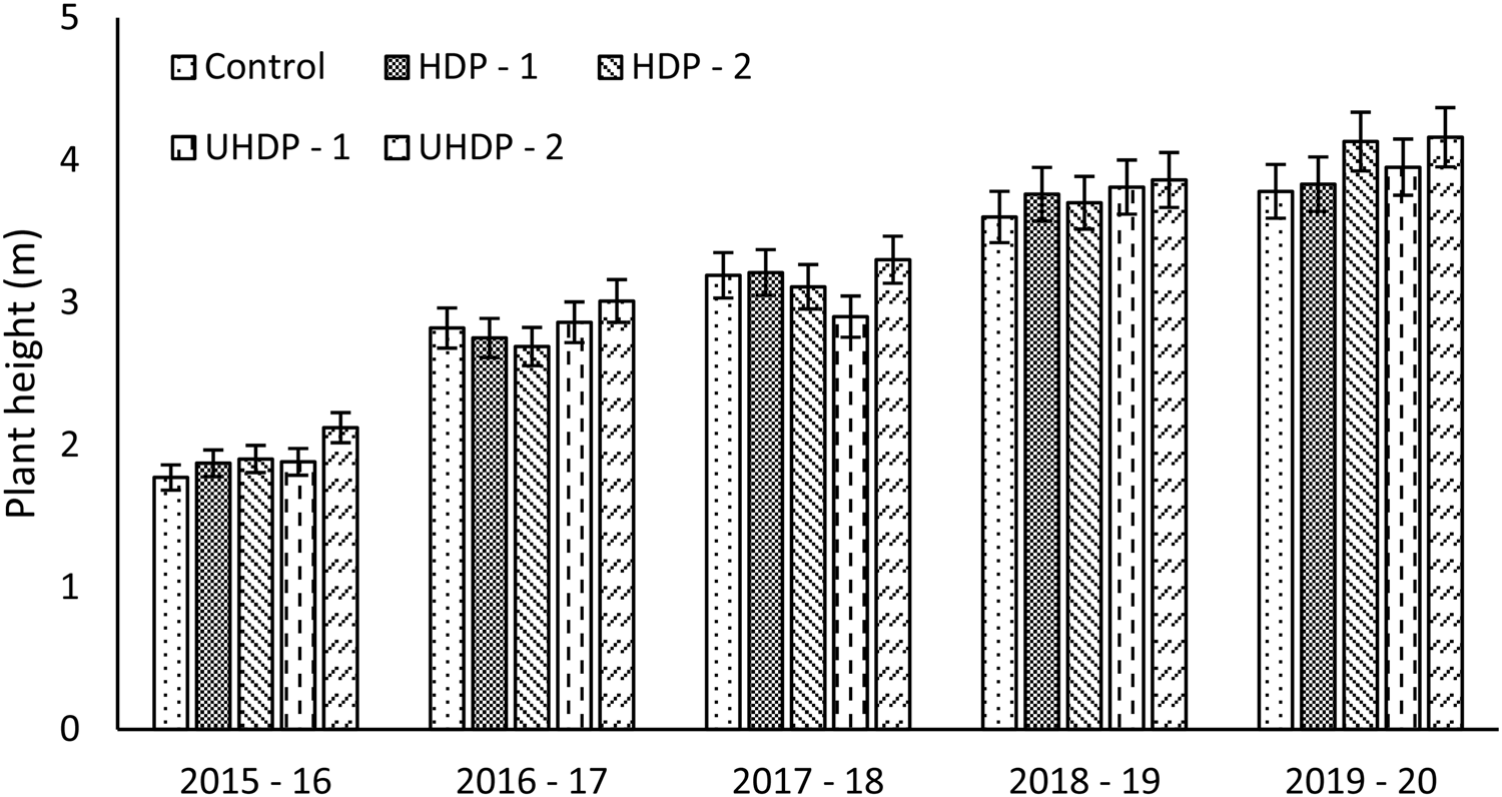
Effect of planting density on height of the plants. Control: Planting at 6 m (row to row) × 6 m (plant to plant) spacing (tree density—277 plants ha^−1^), HDP-1: Planting at 6 m (row to row) × 3 m (plant to plant) spacing (tree density—555 plants ha^−1^), HDP-2: Planting at 4 m (row to row) × 4 m (plant to plant) spacing (tree density—625 plants ha^−1^), UHDP-1: Planting at 4 m (row to row) × 2 m (plant to plant) spacing (tree density—1250 plants ha^−1^), UHDP-2: Planting at 2 m (row to row) × 2 m (plant to plant) spacing (tree density—2500 plants ha^−1^).

Planting densities had significantly influenced plant height, stock girth and canopy volume. Irrespective of the planting densities, the gradual increase in all the growth parameters was observed. Plant height ranged between 1.77 and 4.16 m during the year 2015–16 to 2019–20 and was significantly high in all the treatments over the years (Fig. 1). Plant height decreased with increase in plant spacing. Under conventional spacing, plant height increased gradually. It was found that for want of ample sun light, the plants in close distance grew taller than plants of wide distance plantings. The columnar growth of plants in close spacing was due to poor light interception while plants in wider spacing had lateral and balanced growth due to sufficient space for light interception^15,16^.

**Figure 2:**
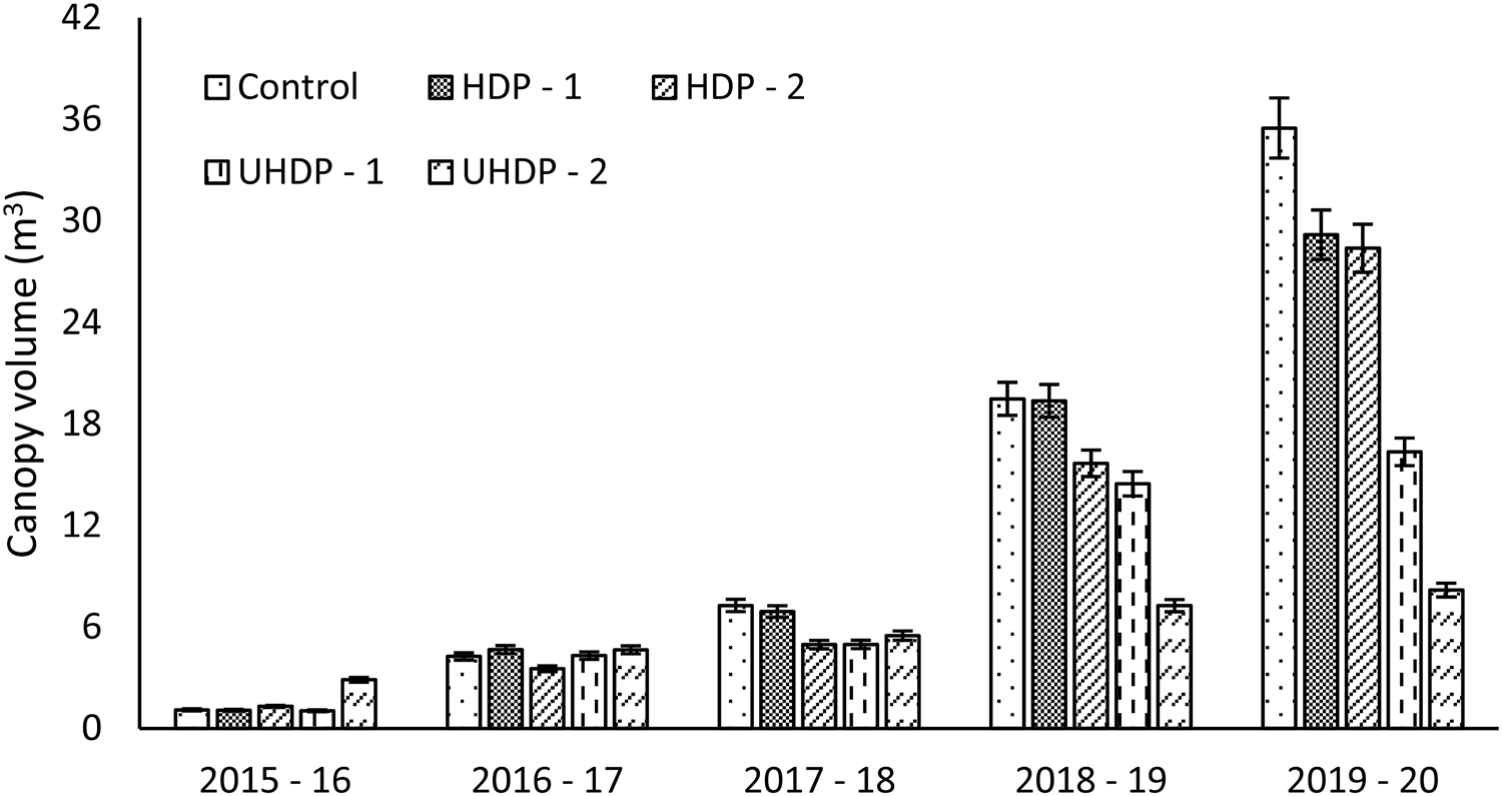
Effect of planting density on canopy volume of the plants. Control: Planting at 6 m (row to row) × 6 m (plant to plant) spacing (tree density—277 plants ha^−1^), HDP-1: Planting at 6 m (row to row) × 3 m (plant to plant) spacing (tree density—555 plants ha^−1^), HDP-2: Planting at 4 m (row to row) × 4 m (plant to plant) spacing (tree density—625 plants ha^−1^), UHDP-1: Planting at 4 m (row to row) × 2 m (plant to plant) spacing (tree density—1250 plants ha^−1^), UHDP-2: Planting at 2 m (row to row) × 2 m (plant to plant) spacing (tree density—2500 plants ha^−1^).

Plant canopy volume is a result of photosynthetically active radiations (PAR), nutrient uptake and irrigation in combination with pests and disease infestation. After third year, large variations were observed in plant canopy volume (1.05–29.18 cu m) (Fig. 2). During fourth year (2015–16), canopy of plants in UHDP-2 system completely occupied the available space and reached to maximum (2.88 cu m). Therefore, spread of all those trees was ceased and subsequent increase observed was also by virtue of the increased plant height. In fourth year itself (2015–16) canopy spread in trees planted at 4 × 2 m spacing peaked in plant to plant direction. However, the maximum canopy volume in conventional system was observed during last year (35.48 cu m). Plants received more light around the canopy at wider spacing and lateral buds proliferated effectively leading to development of lateral branches. Also, enough available space to grow in conventional planting led to obtain spherical canopy. In Kinnow mandarin also the higher growth of plants was observed in conventional planting system (6 × 6 m) than in the high density i.e. 6 × 3 m planting^17^.

**Figure 3:**
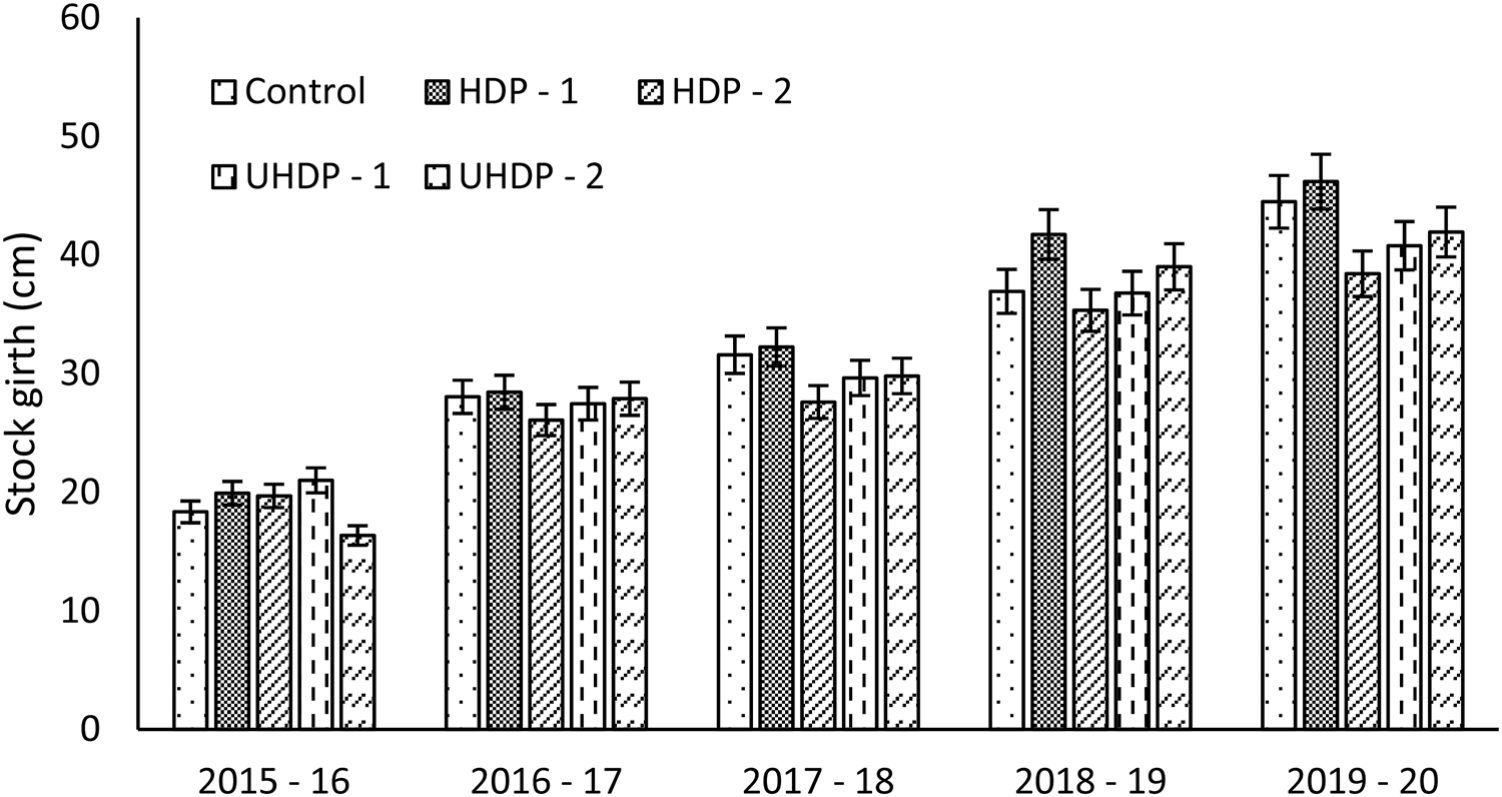
Effect of planting density on stock girth of the plants. Control: Planting at 6 m (row to row) × 6 m (plant to plant) spacing (tree density—277 plants ha^−1^), HDP-1: Planting at 6 m (row to row) × 3 m (plant to plant) spacing (tree density—555 plants ha^−1^), HDP-2: Planting at 4 m (row to row) × 4 m (plant to plant) spacing (tree density—625 plants ha^−1^), UHDP-1: Planting at 4 m (row to row) × 2 m (plant to plant) spacing (tree density—1250 plants ha^−1^), UHDP-2: Planting at 2 m (row to row) × 2 m (plant to plant) spacing (tree density—2500 plants ha^−1^).

In various treatments the average stock girth ranged between 18.34 to 46.18 cm (Fig. 3). However, during initial year the results were non-significant. Plants grown under UHDP-1 (4 × 2 m spacing) recorded maximum stock girth (21.0 cm) during year 2015–16. Thereafter during 2016–17 (28.43 cm), 2017–18 (32.24 cm), 2018–19 (41.73 cm) and 2019–20 (46.48 cm) was maximum under HDP-1 (6 × 3 m spacing). Results revealed no direct relationship between stock girth and canopy volume of the plants. The trees in the conventional planting systems were found to acquire incremental trend of higher stock girth than plants of high and ultra-high density planting. The increased stock girth was to support higher leaf biomass and plant spread of conventional system^16,18^.

### Physiological parameters (Intensity of PAR light, leaf area index and chlorophyll content)

Photosynthetically active radiations (PAR) in terms of photosynthetic photon flux density (PPFD) recorded highly significant variation in all East (97–160), West (96–173), North (78–174) and South (77–136) directions of the plants (Table 2). The highest PAR in all the directions (136–174) was observed in plants grown under control (6 × 6 m spacing). It revealed that there was the lowest solar radiation utilization per unit of land in conventional spacing. Whereas, significantly low intensity of PAR in all directions of plants was found in UHDP-1 (77–101) and UHDP-2 (96–99) spacing treatments. Optimum light interception can be defined as a level of light intercepted by an orchard system above or below which, the economic yield will be reduced^19^. It indicated that there was the highest sunlight utilization per unit area in ultra high density plantings, which could be due to higher coverage of leaf area than control. Plants grown under these treatments facilitated better utilization of solar radiation and increased photosynthetic efficiency of the plant^6^.

**Table 2:** Effect of planting density on PAR light intensity, leaf area index and leaf chlorophyll content (pooled data for 2015–16 to 2019–20).

Treatments	PPDF reading in open sunlight and below the plant canopy in different directions (µ mole^−1^ m^2^ s^−1^)					Chlorophyll content fluorometer readings	Leaf area index
	East	West	North	South	Mean		
Control	160.2 (17.1^a^)	173.6 (18.6^a^)	174.5 (23.3^a^)	136.9 (16.2^a^)	161.29 (31.15^a^)	0.70^a,b^	1.28^a,b^
HDP-1	120.2 (13.8^b^)	129.3 (14.7^b^)	105.6 (13.6^b^)	82.5 (10.2^c^)	109.39 (21.65^b^)	0.71^a^	1.35^a^
HDP-2	101.4 (11.1^c^)	110.4 (12.1^c^)	108.5 (13.7^b^)	107.6 (13.1^b^)	106.97 (20.60^b^)	0.70^a,b^	1.11^c^
UHDP-1	101.1 (11.8^c^)	96.3 (11.1^c,d^)	78.2 (10.4^c^)	77.3 (9.8^c^)	88.20 (17.71^c^)	0.71^a^	1.16^b,c^
UHDP-2	97.0 (10.5^c^)	97.8 (9.7^d^)	96.3 (12.3^b^)	99.3 (12.4^b^)	97.58 (18.58^c^)	0.70^b^	1.09^c^

Avg. PPDF reading in open sunlight was 1250 µ mole^−1^ m^2^ s^−1^.

Figures under parenthesis are transformed values.

Means with at least one letter common are not statistically significant using TUKEY's Honest Significant Difference at 5%

Control: Planting at 6 m (row to row) × 6 m (plant to plant) spacing (tree density—277 plants ha^−1^), HDP-1: Planting at 6 m (row to row) × 3 m (plant to plant) spacing (tree density—555 plants ha^−1^), HDP-2: Planting at 4 m (row to row) × 4 m (plant to plant) spacing (tree density—625 plants ha^−1^), UHDP-1: Planting at 4 m (row to row) × 2 m (plant to plant) spacing (tree density—1250 plants ha^−1^), UHDP-2: Planting at 2 m (row to row) × 2 m (plant to plant) spacing (tree density—2500 plants ha^−1^).

The 6 × 3 m spacing occupied the available space between plant to plant direction (3 m) by the dense and vigorous plant canopy and recorded maximum (1.35) leaf area index (Table 2). The space in the row to row direction (6 m) facilitated light interception that in turn, could have resulted in the higher number of well-developed leaves. Harnessing water, fertilizers, sunlight in this treatment might have translated into increased photosynthesis and thereby higher chlorophyll contents (0.71) than control (Table 2). Plants at UHDP-2 spacing treatment had lowest leaf area index (1.09). Due to narrow spacing, central and intermingling branches were pruned periodically to facilitate open structure for light interception and air circulation. Plants grown under HDP-2 and UHDP-1 spacing recorded intermediate leaf area index and lower chlorophyll content. Whereas, the lowest Chlorophyll contents (0.70) under UHDP-2 spacing (Table 2) could be because of low rate of photosynthesis.

### Leaf nutrient contents

Pooled data showed that leaf N, P and K content significantly varied from 2.32–2.44%, 0.09–0.10% and 1.64–1.79%, respectively and was maximum in the plants spaced at 6 × 3 m (Table 3). Micronutrient contents in the leaves also varied significantly by different treatments of planting. All the nutrients were above optimum range in all the treatments. Pooled data also revealed that leaf Cu, Fe, Mn and Zn contents varied from 11.95–14.3 ppm, 116.7–133.5 ppm, 67.2–76.9 ppm and 15.5–20.51 ppm, respectively (Table 3). Maximum leaf contents of Cu and Mn was observed in 6 × 3 m spacing while Fe and Zn were observed in 4 × 4 m spacing. Improved physical as well as biological health of the soil due to increased organic carbon could have resulted in mobilization of immobile nutrients and helped in increasing their uptake by the plants. Increased nutrient uptake with the increased organic carbon was reported in several fruit crops viz. mandarin^20^, guava^21^, sweet orange^22^ and pomegranate^23^. Almost all the nutrient contents in ultra—high density and high density plantings were low. It was in spite of higher organic carbon contents in these treatments. Periodical pruning and training of the threes could have removed considerable amounts of nutrients in various plant parts. Moreover, substantial amount of nutrients could be required to produce nearly three times more fruit yield per unit area as compared to the plants grown under conventional system of plantation, thereby, reducing nutrient contents in leaf in long run^6^.

**Table 3:** Effect of planting density on leaf nutrient contents (pooled data for 2015–16 to 2019–20).

Treatments	N (%)	P (%)	K (%)	Cu	Fe	Mn	Zn
	(%)			(ppm)			
Control	2.39 (30.2^a^)	0.090 (17.5^b^)	1.64 (23.6^d^)	13.7 (18.9^a,b^)	129.1 (24.5^b^)	68.8 (16.1^c^)	20.1 (10.2^a^)
HDP-1	2.44 (29.9^a^)	0.100 (19.4^a^)	1.77 (26.6^a^)	14.3 (19.7^a^)	116.7 (22.2^c^)	76.9 (18.6^a^)	15.5 (7.6^b^)
HDP-2	2.44 (29.9^a^)	0.100 (18.4^b^)	1.69 (24.9^b,c^)	13.9 (18.2^b,c^)	133.5 (25.9^a^)	74.9 (17.0^b,c^)	20.5 (10.7^a^)
UHDP-1	2.40 (28.2^b^)	0.100 (19.6^a^)	1.79 (25.6^b^)	11.9 (16.9^c^)	132.2 (24.9^a,b^)	75.8 (17.9^a,b^)	20.4 (9.7^a^)
UHDP-2	2.32 (28.1^b^)	0.100 (17.9^b^)	1.68 (24.2^ c,d^)	12.5 (17.5^b,c^)	128.6 (24.6^a,b^)	67.2 (15.7^c^)	19.0 (9.3^a^)

Figures under parenthesis are transformed values.

Means with at least one letter common are not statistically significant using TUKEY's Honest Significant Difference at 5%

Control: Planting at 6 m (row to row) × 6 m (plant to plant) spacing (tree density—277 plants ha^−1^), HDP-1: Planting at 6 m (row to row) × 3 m (plant to plant) spacing (tree density—555 plants ha^−1^), HDP-2: Planting at 4 m (row to row) × 4 m (plant to plant) spacing (tree density—625 plants ha^−1^), UHDP-1: Planting at 4 m (row to row) × 2 m (plant to plant) spacing (tree density—1250 plants ha^−1^), UHDP-2: Planting at 2 m (row to row) × 2 m (plant to plant) spacing (tree density—2500 plants ha^−1^).

### Incidence of insect-pests

Citrus leaf miner, citrus psylla, aphids and mites were observed to be the most important pests of Nagpur mandarin during the different growth phases from 2013 to 2020 (Table 4). The data were collected coinciding with different periods of new flushes during pre-bearing period so as to get exact picture of pest incidence, without interference of fruiting seasons. It was found that citrus leaf miner remained active during all the flushing seasons irrespective of spacing. Maximum incidence of leaf miner was observed in closer spacing of UHDP-2 with mean value of 40.07% over a period of six years while wider spacing of 6 × 6 m recorded significantly lower infestation (18.46%). The psylla population also followed similar trend during the seasons and varied from 27.59 per 5 cm shoot in UHDP-2 to 6.44 per 5 cm shoot in control (Table 4). Pooled mean data indicated that the closer spacing had significantly highest aphid population (30.58 shoot^−1^). Similar trend was also observed in the case of mites (Table 4).

**Table 4:** Effect of planting density on incidence of insect pests (pooled data for 2015–16 to 2019–20).

Treatments	Leaf miner Infestation (%)	Psylla population (per 5 cm twig)	Aphid population (twig^−1^)	Mite infestation (%)
Control	18.46 (24.02^d^)	6.44 (2.45^e^)	8.23 (2.83^e^)	10.67 (19.05^d^)
HDP-1	25.54 (29.57^c^)	9.98 (3.10^d^)	10.58 (3.23^d^)	14.73 (22.55^d^)
HDP-2	31.20 (33.32^b^)	15.02 (3.78^c^)	15.49 (3.90^c^)	20.70 (26.98^c^)
UHDP-1	34.96 (35.77^b^)	20.52 (4.39^b^)	20.66 (4.53^b^)	26.58 (30.99^b^)
UHDP-2	40.07 (39.03^a^)	27.59 (5.10^a^)	30.58 (5.48^a^)	32.63 (34.76^a^)

Figures under parenthesis are transformed values.

Means with at least one letter common are not statistically significant using TUKEY's Honest Significant Difference at 5%

Control: Planting at 6 m (row to row) × 6 m (plant to plant) spacing (tree density—277 plants ha^−1^), HDP-1: Planting at 6 m (row to row) × 3 m (plant to plant) spacing (tree density—555 plants ha^−1^), HDP-2: Planting at 4 m (row to row) × 4 m (plant to plant) spacing (tree density—625 plants ha^−1^), UHDP-1: Planting at 4 m (row to row) × 2 m (plant to plant) spacing (tree density—1250 plants ha^−1^), UHDP-2: Planting at 2 m (row to row) × 2 m (plant to plant) spacing (tree density—2500 plants ha^−1^).

There was significant increase in sucking pests’ infestation and was inversely related to planting distance. It was evident that high density spacings proportionately favored the insect infestation. Narrow planting distance favored the dense crop growth. The augmented favorable microclimate i.e. low temperature, high humidity, less sunlight, which accelerated the new flush growth could have actively helped for insect pests’ multiplication^24,25^. The pruning operation in high density systems accelerates the new growth that attracts leaf miner and the intensity of infestation remains in ratio of area and intensity of pruning^26,27^. It is now established that in citrus, pruning is needed for the high density planting and therefore, it was felt that the tree structure of a hedge of maximum 2.5 m height along with 0.7 m in the base of the trunk and 1 m width that is free of lateral canopy would be needed^28^. Also, to have such desired canopy, customized mechanization should be contemplated on priority. Ultra high density planting system (4.2 × 0.9 m, 4.2 × 1.2 m, 4.2 × 1.5 m, 4.2 × 1.5 and 2.5 × 2.5 m) and high density planting system (5.0 × 2.5 m, 5.0 × 5.0 m and 7.5 × 5.0 m) in mango recorded higher incidence of sucking insect pests like leaf hopper^24,25^. Also, fruit fly damage was comparatively less in conventional planting system 10 × 10 m^24^. Similarly, incidence of thrips, mealybug, aphids were observed significantly high in ultra-high density plantation of pomegranate^29^.

### Fruit yield and quality

**Figure 4:**
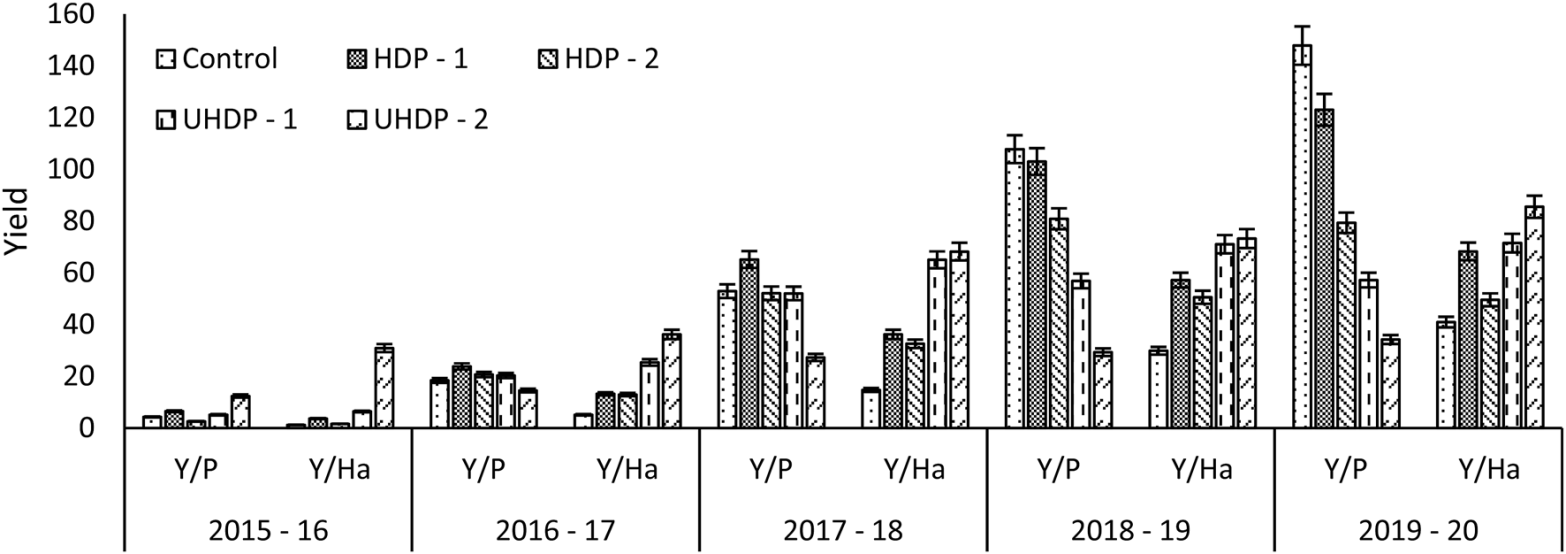
Effect of planting density on fruit yield in terms of per plant and per hectare. Control: Planting at 6 m (row to row) × 6 m (plant to plant) spacing (tree density—277 plants ha^−1^), HDP-1: Planting at 6 m (row to row) × 3 m (plant to plant) spacing (tree density—555 plants ha^−1^), HDP-2: Planting at 4 m (row to row) × 4 m (plant to plant) spacing (tree density—625 plants ha^−1^), UHDP-1: Planting at 4 m (row to row) × 2 m (plant to plant) spacing (tree density—1250 plants ha^−1^), UHDP-2: Planting at 2 m (row to row) × 2 m (plant to plant) spacing (tree density—2500 plants ha^−1^); Y/P: yield plant^−1^ (kg plant^−1^), Y/Ha: yield hectare^−1^ (tons hectare^−1^).

Early fruiting (2015–16) was observed under UHDP-2 (2500 plants ha^−1^). Plants grown under this system attained rapid vegetative growth especially plant height and led to induction of flowering and fruiting. Commercial yield obtained from 2015–16 onwards was found to vary from 14.5 to 147.8 kg plant^−1^. The highest yield under 6 × 3 m spacing during 2016–17 was 23.8 kg plant^−1^ and during 2017–18 was 65.1 kg plant^−1^ (Fig. 4). Afterward, the highest yield per plant was observed in conventional 6 × 6 m spacing treatment. The canopy volume of the plants under this treatment was increasing, while the growth restricted high density systems as plants occupied almost all the allotted space. As such, significantly lowest per plant yield was obtained in close spacing plants as compared to conventional spacing treatments.

However, on area basis, there was vast difference in fruit yield (1.2–85.5 t ha^−1^) amongst the treatments (Fig. 4). Under UHDP-2 treatment, fruit yield (36.2 t ha^−1^) was more than six folds as compared to conventional 6 × 6 m (5.1 t ha^−1^) during 2016–17. The difference in yield on area basis amongst treatments became narrow over the years and during 2019–20 the yield was just two folds more in the UHDP-2 than conventional treatment. The increase in plant density increased the yield per unit area. Better utilization of solar radiation, increased nutrients, water and photosynthetic efficiency facilitated the increase in yield. As such under UHD, the largest amount of fruit bearing area per unit volume could be attained if the trees are planted as narrow hedges at about 1.8 m spacing with tree height and distance between rows adjusted to allow sufficient light^4,6^. Higher yields in close spacing confirms the findings of Nawaz et al*.*^16^, Wheaton et al*.*^30^ and Azevedo et al*.*^31^ found that there is an inverse relationship between tree spacing and yields. Closely spaced trees were reported to harness higher amount of solar radiations to synthesize photosynthates, which directly contribute to the growth, higher number of leaves, branches that proved beneficial towards increased sink strength resulting in higher yield in Kinnow^32^. However, moderate densities than higher density of fewer than 1000 trees per ha were preferred for Florida conditions^33^.

The fruit quality parameters showed that various spacing treatments significantly influenced quality of fruits in terms of fruit breadth (72.58–75.61 cm), fruit length (65.35–69.69 cm), peel thickness (2.65–2.90 mm), juice content (43.08–45.71%), total soluble solids (TSS) contents (9.15–9.57^0^Brix) and juice acidity (0.75–0.81%) while, TSS:acidity ratio in fruit juice showed non-significant variation (Table 5). Fruit length and fruit breadth were significantly higher in the fruits of the plants grown at normal spacing of 6 × 6 m (control); however, fruit weight was non-significant. Fruit weight is a function of number polygenic characters viz. of juice content, peel thickness, number of seeds etc. Hence, the size of fruits could not only alter the fruit weight. Similar results were obtained in Kinnow where bigger size fruits with higher juice contents were recorded at 5 × 4 and 5 × 5 m spacings^34^. Fruits with thin peel were produced under UHDP-2. Fruit TSS and juice acidity were higher in HDP-1 and HDP-2, respectively. Light canopy thinning every year promoted penetration of sunlight in UHDP-2 resulting in leaves, shoots and good quality fruit development. Klerk^35^ observed that higher rate of transpiration and respiration produce more carbohydrates when leaves are exposed to sunlight. Increased fruit yield without compromising quality under high density planting of fruit crops were reported by Mishra and Goswami^36^. Albeit, Gaikwad et al*.*^37^ could not find any improvement in fruit quality of mango by reducing planting distance. After harvest, skirting and light hedging was recommended as correctly skirted trees appear to “reallocate” flowering and fruiting to remaining parts of the canopy^38,39^. Findings indicate that plant density and prunning practices for canopy management have certain effect on fruiting and fruit quality that warrants detailed further study.

**Table 5:** Effect of planting density on fruit quality parameters (pooled data for 2015–16 to 2019–20).

Treatments	Fruit weight (g)	Fruit length (mm)	Fruit breadth (mm)	Peel thickness (mm)	Fruit Juice content (%)	TSS (^0^Bxix)	Titrable acidity* (%)	TSS: acid ratio
Control	145.23	69.69 (34.68^a^)	75.61 (42.40^a^)	2.87 (32.73^b^)	45.71 (30.77^a^)	9.15 (37.67^b^)	0.78 (17.47^a,b^)	12.19 (15.72)
HDP-1	146.25	65.36 (32.99^b,c^)	73.57 (41.27^b^)	2.79 (32.70^b^)	45.33 (30.60^a^)	9.57 (39.20^a^)	0.75 (17.24^a,b^)	13.16 (16.37)
HDP-2	144.26	66.03 (33.25^b,c^)	72.82 (40.98^b^)	2.90 (34.28^a^)	43.61 (29.36^b^)	9.50 (39.15^a^)	0.81 (17.89^a^)	12.34 (15.76)
UHDP-1	143.89	67.28 (33.47^b^)	72.71 (40.67^b^)	2.74 (32.31^b^)	43.08 (29.02^b^)	9.21 (38.53^ab^)	0.77 (17.11^ab^)	12.83 (15.93)
UHDP-2	144.37	65.35 (32.56^c^)	72.58 (40.51^b^)	2.65 (32.55^b^)	45.46 (30.33^a^)	9.22 (38.14^b^)	0.75 (16.80^b^)	12.95 (16.06)

Figures under parenthesis are transformed values.

Means with at least one letter common are not statistically significant using TUKEY's Honest Significant Difference at 5%

Control: Planting at 6 m (row to row) × 6 m (plant to plant) spacing (tree density—277 plants ha^−1^), HDP-1: Planting at 6 m (row to row) × 3 m (plant to plant) spacing (tree density—555 plants ha^−1^), HDP-2: Planting at 4 m (row to row) × 4 m (plant to plant) spacing (tree density—625 plants ha^−1^), UHDP-1: Planting at 4 m (row to row) × 2 m (plant to plant) spacing (tree density—1250 plants ha^−1^), UHDP-2: Planting at 2 m (row to row) × 2 m (plant to plant) spacing (tree density—2500 plants ha^−1^).

*Titrabile acidity as citric acid.

### B:C ratio

Though fruit yield was obtained during fourth year of plantation (2015–16), monetary returns were less than the cumulative expenditure incurred towards initial plantation and yearly maintenance cost. B:C ratio was below one (0.18–0.93) in various treatments but it was close to 1 (0.93) at UHDP-2 at the first harvest itself (Table 6). At UHDP-2 yield was 15 times more than the conventional system (6 × 6 m) and benefit with respect to cost was equal. During next year (2016–17), except conventional method of plantation, profits were obtained under all HDP and UHDP treatments as indicated by B:C ratio, which was above one and the highest (3.14) was under UHDP-2. During sixth year after plantation (2017–18) the lowest profit (B:C ratio 2.38) was recorded under control and highest (B:C ratio 5.26) in UHDP-2. At the end of the experiment (2019–20), the highest B:C ratio was recorded in 6 × 3 m (B:C ratio 6.36) followed by 4 × 2 m (B:C ratio 6.09) and 2 × 2 m (B:C ratio 6.04) spacing treatment. It was well evident that the increased cost of cultivation in high density planting systems than control was due to additional cost incurred for field preparation to accommodate higher number of plants per unit area and increased plant population. Therefore, although there were higher gross returns in high density plantings during initial harvests than control, it could not be translated in net returns. Wheaton et al*.*^33^ was of opinion that there was little advantage in high tree density (2020 trees ha^−1^) than moderate densities of fewer than 1000 trees ha^−1^ in Hamlin and Valencia oranges, ‘Murcott’ tangor and ‘Redblush’ planted on 15 rootstocks and own-rooted cuttings under high density planting. In Afourer mandarin and Navel orange, high density planting (952 plants ha^−1^ or 600 plants ha^−1^) had higher investment initially but they produced higher yields in the early years and could pay-off the debt resulting in quicker cash flow breakeven, two years earlier than traditional (440 plants ha^−1^) density^40^.

**Table 6:** Effect of planting density on benefit: cost (B:C) ratio.

Treatments	2015–16	2016–17	2017–18	2018–19	2019–20
Control	0.18	0.69	2.38	3.76	5.32
HDP-1	0.38	1.45	5.2	5.09	6.36
HDP-2	0.16	1.1	5.06	4.95	5.66
UHDP-1	0.39	1.6	4.96	5.34	6.09
UHDP-2	0.93	3.14	5.26	5.5	6.04

Control: Planting at 6 m (row to row) × 6 m (plant to plant) spacing (tree density—277 plants ha^−1^), HDP-1: Planting at 6 m (row to row) × 3 m (plant to plant) spacing (tree density—555 plants ha^−1^), HDP-2: Planting at 4 m (row to row) × 4 m (plant to plant) spacing (tree density—625 plants ha^−1^), UHDP-1: Planting at 4 m (row to row) × 2 m (plant to plant) spacing (tree density—1250 plants ha^−1^), UHDP-2: Planting at 2 m (row to row) × 2 m (plant to plant) spacing (tree density—2500 plants ha^−1^).

## Conclusions

The present study attempted to ascertain effects of various plant densities on horticultural, physiological and fruit quality parameters and yield of Nagpur mandarin along with B:C ratio. It was possible to manage the canopy of plants planted under extremely close spacing of 2 × 2 m (2500 plant ha^−1^) and translate it into getting early and high yields. The yield obtained under 2 × 2 m spacing treatment was twenty-six times higher than control from the very first fruiting. Albeit, monetary returns were not translated into net profitability during first year due to very high initial cost incurred on this treatment. Higher returns with increasing B:C ratio was obtained under this treatment from 2nd to 4th years of fruiting. In 5th year 6 × 3 m spacing treatment (555 plants ha^−1^) was found economically superior. The management practices helped to retain the quality of fruits and yield which could not be adversely affected by increased insect—pest and disease infestation by virtue of close planting. It is important to observe performance of these plantations further for next 4–5 years from commercial point of view. This study has established practical feasibility and economic superiority of high and ultra-high density planting of Nagpur mandarin over conventional system of plantation. In high density plantation technique, canopy management is equally important in bearing citrus trees to have better interception of PAR and aeration, strong sap flow for fruiting and supporting branches for convenient harvesting. Last but not the least, effective alternative to harness available space for increasing net return for first few years of fruiting is high density planting. However, the synchronization of spacings vis-à-vis growth pattern needs to be standardized and mechanization for canopy management would be an area for future work to ascertain optimum economic returns.

## Materials and methods

### Experimental site

Present study was conducted at Mohpa village in Kalmeshwar Taluka of Nagpur District, Maharashtra, India in farmer’s field (330 m altitude, 21° 23′ 22″ N latitude, 78° 92′ 23″ E longitude). The experiment site was bestowed with hot and dry summer followed by mild winter and average temperature range of 26.32–41.80 °C and 9.35–27.30 °C, respectively. Relative humidity at site ranged between 18.94 and 87.97%, along with about 1125 mm average annual precipitation during the months of June–September mostly through monsoon rains.

Very deep soil of the experimental field had montmorillonite mineralogy. Textural and physico-chemical properties of the soil were: sand 15.9%, silt 16.8%, coarse fragments (> 2.0 mm) 4.2%, clay 67.3%. Soil EC (0.11 dS m^−1^), pH (7.84), calcium carbonate (12.3%) and organic carbon (0.55%) were in optimum range for mandarin production. Nutrient contents status was: N (338.6 kg ha^−1^), P (22.6 kg ha^−1^) and K_2_O (293.6 kg ha^−1^), while available Fe, Mn, Cu and Zn were 12.9, 16.5, 3.15 and 0.56 ppm, respectively. Soil had field capacity 36.3% (33 kPa) and permanent wilting point 18.7% (1.5 M Pa).

### Experimental setup

The experimental study was started in 2012 by plantation of one-year-old Nagpur mandarin budlings raised on Rangpur lime rootstock at various spacings at farmer’s field at Mohapa village, district Nagpur, Maharashtra, India. Plantation was maintained as per standard practices^41^. Data on various parameters were analyzed from 2014–15 to 2019–20. A randomized block design was used for laying experiment in the field wherein five treatments were replicated 4 times having 10 plants per replication. The plants were raised through standard training and pruning practices. The various treatments were as follows, Control**:** Conventional planting at 6 m (row to row) × 6 m (plant to plant) spacing (tree density—277 plants ha^−1^), High density planting-1 (HDP-1)**:** Planting at 6 m (row to row) × 3 m (plant to plant) spacing (tree density—555 plants ha^−1^), High density planting-2 (HDP-2)**:** Planting at 4 m (row to row) × 4 m (plant to plant) spacing (tree density—625 plants ha^−1^), Ultra high density planting-1 (UHDP-1): Planting at 4 m (row to row) × 2 m (plant to plant) spacing (tree density—1250 plants ha^−1^), Ultra high density planting-2 (UHDP-2): Planting at 2 m (row to row) × 2 m (plant to plant) spacing (tree density—2500 plants ha^−1^). Data on various parameters were recorded randomly from experimental plants.

### Crop husbandry

Plants having uniform growth were selected from nursery and planted in main field as per envisaged treatments. Soil dug out from pits was mixed with farmyard manure (20 kg per pit) and single super phosphate (1 kg per pit) before planting of budlings. First year’s nutrient application was at the rate of 1 kg vermicompost, 150 g N, 50 g P and 25 g K per plant which was doubled for second year and tripled for third year. Nutrient application was fixed at 2 kg vermicompost, 600 g N, 200 g P and 200 g K per plant from fourth year onwards. Nutrient dose was applied through fertigation using diammonium phosphate, urea, muriate of potash and phosphoric acid fertilizers. For application of micronutrients, foliar sprays were applied on fully expanded leaves during new flush seasons i.e. July–August and February–April.

Field was irrigated on alternate days using drip irrigation at cumulative irrigation as equivalent to 0.70 E-Pan with 90% irrigation efficiency of drip. Balance sheet method was used to calculate the effective rainfall from the actual rainfall received. Accordingly, daily water requirement was met out.

Standard package of practices was adopted for the control of various insect—pests and diseases^41^. During initial years, plants were raised under various treatments to provide proper shape. While training, branches were oriented in such a manner that the plant canopy at central place would facilitate better light penetration and distribution. Up to 75 cm from ground no shoots were allowed to grow. Thereafter 3–4 main scaffold branches were grown followed by secondary and tertiary branches. At 2 × 2 m spacing (UHDP-2), between two rows, duct space of 60 cm was created for proper access after 3rd year with light pruning. This light canopy thinning was required in this treatment every year after third year. Dried twigs were removed after harvesting every year followed by fungicidal sprays.

### Soil physico-chemical properties and fertility status

Every year, the composite soil samples from 30 cm soil depth (45 cm away from the drip line of the tree) and weighing up to 1 kg were collected before fruiting from each treatment and analyzed for various chemical properties and fertility status. Analysis of soil organic carbon^42^, available N^43^, available P^44^ and available K^45^ were determined by standard procedures.

### Plant growth and physiological parameters

Plant growth parameters namely, plant height, plant spread (East–West and North–South direction), stock and scion girth were recorded 15–30 cm above the ground level, in the month of February each year.

The index of the leaf area in plants for seasonal variations in growing phase was measured by Digital Plant Canopy Imager (Make, CID, Bio-science). Chlorophyll Fluorometer (Make WALZ Junior Pam) was employed for measurement of Leaf chlorophyll contents and expressed as SPAD values. Photosynthetically Active Radiation (PAR) expressed as Photosynthetic Photon Flux Density (PPFD) were measured by Quantum Light Meter (Spectrum Technologies Inc., USA make). On clear sunny days the light meter was held horizontally under the pre-selected plants in North, East, West, and South directions with sensor center positioned 50 cm inside the tree drip line (shadow of tree canopy) at mid-day.

### Leaf nutrient contents

Leaf samples (5–7 months old leaves) were collected every year from non-bearing shoots of each selected plant in each treatment^46^ and analyzed for different macro- and micro-nutrient status. Samples were washed thoroughly in water, liquid soap, acidic water and glass redistilled water sequentially, air dried followed by oven drying at 70 °C till samples maintained constant weight. Composite leaf samples were used for analyzing total macro (N, P and K) and micronutrients (Fe, Zn, Mn and Cu) using standard methods described in Chapman and Pratt^47^.

### Incidence of insect-pests

Incidence of insect pests of citrus viz. citrus leaf miner (*Phyllocnistis citrella* Stainton), Asian Citrus Psylla (*Diaphorina citri* Kuwayama), aphids (Aphis sp., *Toxoptera *sp.) and red spider mites (*Eutetranychus orientalis* Klein) were recorded during flushing seasons i.e. *Ambia* (January–February), *Mrig* (June–July) and *Hasta* (September–October) at fortnightly intervals. Four twigs of 15–20 cm length from North, South, East and West direction of each tree were observed and likewise 10 replications were taken to calculate infestation (in case of citrus leaf minor and mites). Population count per 5–10 cm twig for psylla and aphids and counts per tapping for thrips was taken. Similarly trap catch of citrus leaf miner adult males using sex pheromone lure in a delta trap^48^ were also installed for monitoring the field population during flushing season.

### Fruit quality and yield

The fruit yield data from 2015–16 to 2019–20 was recorded on tree basis (kg tree^−1^) and area basis (t ha^−1^). Also, physico-biochemical properties of fruit viz. Total soluble solids (TSS), acidity, juice content and peel thickness were recorded with standard procedures^49^.

### B:C ratio

Benefit:Cost (B:C) ratio was calculated out to unravel economic viability of the technology. The cost incurred towards land preparation, pit digging, irrigation and budling (budded seedlings with selected scion bud by shield budding) during first year was Rs. 55,942, 73,984, 85,281, 1,29,812 and 2,10,875 in planting at 6 × 6 m, 6 × 3 m. 4 × 4 m, 4 × 2 m and 2 × 2 m spacing. It cumulatively increased to Rs. 1,63,875, 2,34,751, 2,45,331, 4,05,662 and 8,33,325, respectively until fruit bearing stage (fourth year). B:C ratio was calculated for treatments over conventional planting system of 6 × 6 m. spacing. The B:C ratio greater than one (> 1) represents profit, as the benefit will be more than the cost incurred.

### Statistical analysis

The present study which had five treatments replicated four times, was conducted in randomized block design. Significant differences among various treatments during the years of experimentation were pooled for statistical analysis. Tukey’s HSD test was done by using SAS software for shortest significant range tests^50^.

### Declaration

Experimental research and field studies on plants (either cultivated or wild), including the collection of plant material, were complied with relevant institutional, national, and international guidelines and legislation.

### Ethics

The permission to use plants used in this experiment has been obtained from an appropriate governing body.

### Compliance with ethical standards

All the ethical standards have been followed.
